# Predicting Efficacy of a Purified Inactivated Zika Virus Vaccine in Flavivirus-Naïve Humans Using an Immunological Correlate of Protection in Non-Human Primates

**DOI:** 10.3390/microorganisms12061177

**Published:** 2024-06-11

**Authors:** Camilo J. Acosta, Francesco Nordio, David A. Boltz, Whitney R. Baldwin, Greg Hather, Eloi Kpamegan

**Affiliations:** Takeda Vaccines Inc., Cambridge, MA 02142, USA; francesco.nordio@takeda.com (F.N.); david.boltz@takeda.com (D.A.B.); whitney.baldwin@takeda.com (W.R.B.); ghather@gmail.com (G.H.); eloi.kpamegan@takeda.com (E.K.)

**Keywords:** Zika virus, surrogate endpoint, correlate of protection, purified inactivated Zika vaccine, PIZV, TAK-426, non-human primates

## Abstract

A traditional phase 3 clinical efficacy study for a Zika vaccine may be unfeasible because of the current low transmission of Zika virus (ZIKV). An alternative clinical development approach to evaluate Zika vaccine efficacy (VE) is therefore required, delineated in the US FDA’s Accelerated Approval Program for licensure, which utilizes an anti-Zika neutralizing antibody (Zika NAb) titer correlated with non-human primate (NHP) protection as a surrogate endpoint. In this accelerated approval approach, the estimation of VE would be inferred from the percentage of phase 3 trial participants achieving the established surrogate endpoint. We provide a statistical framework to predict the probability of protection for human participants vaccinated with a purified inactivated ZIKV vaccine (TAK-426), in the absence of VE measurements, using NHP data under a single-correlate model. Based on a logistic regression (LR) with bias-reduction model, a probability of 90% protection in humans is expected with a ZIKV NAb geometric mean titer (GMT) ≥ 3.38 log_10_ half-maximal effective concentration (EC_50_). The predicted probability of protection of TAK-426 against ZIKV infection was determined using the two-parameter LR model that fit the calculated VE in rhesus macaques and the flavivirus-naïve phase 1 trial participants’ ZIKV NAb GMTs log_10_ EC_50_, measured by a ZIKV reporter virus particle assay, at 1 month post dose 2. The TAK-426 10 µg dose predicted a probability of protection from infection of 98% among flavivirus-naïve phase 1 trial participants.

## 1. Introduction

It is estimated that half of the global population lives in regions that have reported Zika virus (ZIKV) cases [1]. After the large outbreak in the Americas in 2015–2016 [2], ZIKV has continued to circulate at low and sustained levels in the Americas [3] and Southeast Asia [4,5,6], together with locally acquired ZIKV cases in the United States [7] and France [8] and ZIKV outbreaks in India [9,10]. Given the significant threat to public health due to complications associated with ZIKV, especially to children born to women infected with ZIKV during pregnancy, the development of a vaccine is crucial to protect susceptible populations, including travelers [11] visiting Zika-endemic countries [1,12].

Although ZIKV infection during pregnancy is typically asymptomatic or is characterized by mild clinical symptoms, it has been associated with serious outcomes for fetuses and newborns. Congenital ZIKV syndrome (CZS) includes microcephaly, fetal death, placental insufficiency, fetal growth restriction, and severe neurological disorders [13,14,15,16]. ZIKV infection is also associated with Guillain-Barré syndrome [17,18,19,20,21,22,23,24] and other neurological conditions in adults [18,25,26,27]. In addition, severe disease requiring hospitalization due to ZIKV infection has been reported, but is uncommon with rare cases of death outcome; such deaths have been primarily reported in immunocompromised individuals or patients with other co-morbidities [28,29].

A traditional phase 3 clinical efficacy study of a Zika vaccine may not be feasible due to the low transmission rates of ZIKV, its typical asymptomatic presentation, and the unpredictability of future outbreaks. An alternative clinical development approach is therefore required to evaluate vaccine efficacy (VE), delineated in the US Food and Drug Administration’s Accelerated Approval pathway for licensure. Several vaccines are being developed following the accelerated approval process including IXCHIQ (chikungunya), Prevnar 20 (pneumococcal), and Gardasil 9 (human papillomavirus) [30]. In summary, an immunological marker is identified as a surrogate endpoint that is reasonably likely to predict clinical benefit and therefore is utilized to estimate VE. TAK-426-induced anti-Zika neutralizing antibody (Zika NAb) geometric mean titer (GMT), measured by a ZIKV reporter virus particle (RVP) assay, has been shown to correlate with protection in non-human primates (NHPs) [31]. In this accelerated approval approach, the estimation of VE is inferred from the percentage of Phase 3 trial participants achieving the previously established surrogate endpoint: the ZIKV neutralizing antibody titer (Zika NAb) threshold that correlated with protection in NHPs.

The use of ZIKV NAbs as a surrogate endpoint for protection against ZIKV infection is supported by (but not limited to) the following precedents: (i) NAbs specifically directed against the E protein have been identified as correlates of protection for vaccines directed against other flaviviruses (FVs), such as Japanese encephalitis virus, yellow fever virus, and tickborne encephalitis viruses [32]; (ii) different vaccine platforms (DNA, RNA, inactivated virus, protein subunit, adenovirus-vectored, virus-like particle) have been proven to induce ZIKV-specific NAbs that confer protection against ZIKV challenge in animal models [33,34,35,36,37,38,39,40,41]; (iii) passive transfer of monoclonal antibodies or antisera from vaccinated humans and animals to mice or macaques protected against ZIKV challenge [33,39,40,42]; (iv) the licensure pathways for anthrax, Ebola, and chikungunya vaccines were based on extrapolation of a reasonable prediction of human efficacy from animal efficacy studies [43,44,45].

Animal models are adequate to estimate or confirm NAb titer thresholds that correlate with protection against ZIKV infection (i.e., VE) [46]. The closest ZIKV infection model to humans is NHPs. Rhesus and cynomolgus macaques have been used extensively to study the pathogenesis of ZIKV isolates and to determine VE and correlates of protection against ZIKV infection [34,35,36,39,40,46,47,48]. VE against ZIKV vertical transmission has also been evaluated in the pregnant marmoset model [49]. NHP infection models have comparable features to human ZIKV infection, such as susceptibility to infection by different ZIKV lineages, ZIKV viremia kinetics, and the immunological response to Zika vaccines [50,51,52].

Takeda’s investigational purified inactivated ZIKV vaccine (PIZV or TAK-426) is an aluminum hydroxide-adjuvanted vaccine candidate made from purified formalin-inactivated ZIKV. The virus seed was derived from a plaque-purified sub-isolate of ZIKV strain PRVABC59, which was isolated from the 2015 outbreak in the Americas [53]. We conducted a series of in vivo animal studies to evaluate the safety and immunogenicity of two doses of vaccine administered via the intramuscular route with a 28-day interval, as well as protection against experimental ZIKV challenge. Immune responses to TAK-426 have been demonstrated after vaccination of CD1 and AG129 mice (interferon-α/β and interferon-γ receptor knockout mice) [53], New Zealand White rabbits (unpublished), and NHPs (rhesus macaques) [54,55]. TAK-426 has also been shown to be efficacious in AG129 mice [53] and NHPs [55,56]. In a phase 1 trial, TAK-426 demonstrated an acceptable safety profile and was immunogenic in both FV-naïve and FV-primed adults [56], with extended safety follow-up and persistence of ZIKV NAb responses up to 2 years post dose 2 [57]. This report describes the strategy, the initial proof-of-concept methods, and analyses applied to extrapolate a reasonable prediction of TAK-426 VE in humans (FV-naïve adults), based on results from a NHP VE study and a phase 1 study investigating human humoral immune responses.

## 2. Materials and Methods

### 2.1. Source Data Studies

The source data (NHP VE and human phase 1 trials) and detailed research methods to obtain the data used in the following statistical framework are described elsewhere. [54,56]. The TAK-426 clinical trial was a large (N = 271) phase-1 individually randomized observed-blind placebo-controlled study, and the NHP was a randomized observed-blinded placebo-controlled sex and weight-stratified experiment.

### 2.2. Comparing NHP and Human ZIKV NAb GMTs

The testing of all (NHP and human) serum samples was performed with the same ZIKV RVP NAb assay, which is species-independent and employs identical reagents for human and rhesus macaque serum matrices (method described in Bohning et al. 2021) [31]. Standardization of critical vaccination parameters among the phase 1 trial and NHP VE study was followed, including the use of the same vaccine lot, vaccination dosage and regimen, and sample collection schedules.

For both the NHP (FV-naïve) and human (FV-naïve only) NAb data at study day 29 (1 month post dose 1) and 57 (1 month post dose 2), the GMTlog_10_ half-maximal effective concentration (EC_50_) was computed for each dose group and time point separately. The corresponding 95% confidence intervals (CIs) were computed based on the t-distribution. This CI calculation assumed that the NAb GMT values are log-normally distributed within each dose group and time point for both human participants and NHPs.

### 2.3. Reference Definitions

Correlate: Biomarker that (for the moment, at least) has a strong mathematical relationship with the clinical outcome [58].

Surrogate: Biomarker that is established to be associated with clinical protection (human data are required) [58].

### 2.4. Modeling the Relationship between NHP ZIKV NAb GMTs and Zika Viral Load

A two-parameter logistic model (binary outcome) was fitted with data from NHPs to determine the TAK-426-induced correlate of protection. The two parameters were TAK-426 vaccine protection against ZIKV infection and Zika NAb titers. Zika viral RNA (vRNA) copies/mL, determined by RT-qPCR assay, was computed for each dose and timepoint post-ZIKV challenge (study days 71–81 and 84). NHPs were considered protected if Zika vRNA was not detected or was below the assay lower limit of quantitation (LLOQ) for all timepoints; peak Zika vRNA for each macaque was defined as the highest observed vRNA concentration across all timepoints tested. Zika vRNA was measured by RT-qPCR LLOQ). Zika NAb titers (log_10_ EC_50_) were measured by RVP on study day 71. A 2-parameter logistic model was fit using a standard logistic function with logit link of the following form:

$$g\left(\mu \right)=ln\left(\frac{\mu }{1-\mu }\right)=\alpha +\beta X$$ (1)

where *α* and *β* are parameters and *X* is the Zika NAb titers (log_10_ EC_50_). NHPs administered with all vaccine doses (including placebo referred to as 0.000) were used for the analysis. The data was fitted using the R package brglm (version 0.7.2), which fits binomial-response generalized linear models using the bias-reduction method developed by Firth [59].

CIs for the parameters of the original logistic regression (LR) model (without dose as a covariate) were computed using the variance covariance matrix for the parameter estimates. For a given parameter, the 95% CI was calculated as the parameter estimate plus or minus z_0.975_ times the square root of the corresponding diagonal element of the variance covariance matrix. Here, z_0.975_ is the 0.975 quantile of the standard normal distribution.

Two goodness-of-fit methods were applied to assess the LR model: the Hosmer–Lemeshow goodness-of-fit test [60] and the area under the curve using the receiver operating characteristic curve. The Hosmer–Lemeshow test involved dividing the data points into groups according to their predicted probabilities. For this analysis, fewer groups were used due to the small number of NHPs. The Chi-square statistic for each group was summed, and the result was the Hosmer–Lemeshow test statistic, which was expected to have an approximate Chi-square distribution with 8 degrees of freedom. The area under the curve of the receiver operating characteristic curve would describe the sensitivity and specificity for predicting the outcome (protected/not protected) based on the estimated probability of protection.

The ZIKV NAb GMTs that yielded selected probabilities of protection (70% and 90%) were computed by inverting the logistic model. For 90% protection, the CI for the corresponding GMT was calculated as follows: using the variance covariance matrix for the parameter estimates of the logistic model, parameters were simulated from a normal distribution 10,000 times. For each instance of the simulated parameters, the level of the log_10_ NAb GMT log_10_ EC_50_ that gave 90% probability of protection was determined. The 0.025 and 0.975 quantiles from the distribution of these levels were used as the 95% CIs.

### 2.5. The Prentice Criteria

The Prentice Criteria [61] were applied to demonstrate the ability of the ZIKV NAb GMTs from the NHP VE study to serve as a surrogate endpoint to predict VE (protection). The criteria and statistical models are listed in Table 1.

To verify the first Prentice criterion, the protection status (0 = non-protected; 1 = protected) in NHPs was established based on the log_10_ Zika vRNA level defined as the response and the log_10_ TAK-426 dose as the independent variable. An animal was considered protected if the vRNA level was below the lower limit of quantitation for all time points tested. To verify the second criterion, a simple linear regression was fitted to test if the vaccine dose was significantly related to ZIKV NAb GMTs. To verify the third criterion, a two-parameter logistic model (see Equation (1) above) was fitted. Finally, to assess the fourth Prentice criterion (the full effect of the vaccine on protection is explained by ZIKV NAb GMTs, i.e., the base model, Equation (1)), two models, including both ZIKV NAb GMT and TAK-426 dose as covariates, were fitted to the data. The first of these models used TAK-426 dose without transformation:

$$g\left(\mu \right)=ln\left(\frac{\mu }{1-\mu }\right)=\alpha +\beta X+\gamma (PIZV\;dose)$$

The second of these models, described by Fay et al. [62], used the log-transformed TAK-426 dose. To avoid taking the log of a zero dose, the dose for the control group was set to 10^−8^ µg. This value was chosen because it is non-zero but still resulted in an estimated probability of protection that was very close to zero, thus fitting the observed data well. The Akaike information criterion (AIC) [63] values were compared for these two models and the original model. Any model with an AIC value greater than the original model was not included in further analyses, as a greater AIC value is indicative of a poorer fit. The second of these models differed from the original model by an additional additive term, where log dose was used as a covariate. This model had an AIC value of 20.1, whereas the original model had an AIC value of 16.4. Because the original model had the lowest AIC value, the other models were not pursued further.

### 2.6. Estimating the Human Probability of Protection for Each Dose Group

To extrapolate a reasonable prediction of VE in humans from the NHP VE study and the FV-naïve phase 1 trial participants’ immune responses, we considered recommendations from review articles [64,65] and non-clinical studies with Ebola and anthrax vaccines [43,44]. Following the bridging strategy by Yellowlees et al. [66], the predicted probability of protection of the TAK-426 10 µg dose against ZIKV infection was determined. The model used is described above in the section “Modeling the Relationship Between NHP ZIKV NAb GMTs and Zika Viral Load”: a two-parameter LR model that fitted the VE in NHPs (dependent variable) versus the NAb GMTs in NHPs (independent variable). The FV-naïve phase 1 trial participants’ ZIKV NAb GMTs (log_10_ EC_50_) at 1 month post dose 2 (day 57) were used as the independent variable in the same model to project the VE in humans. The probability of protection was estimated for each human participant based on the two-parameter logistic model for NHP protection described in the previous section. The mean probability of protection was then calculated for each dose group.

A bootstrap analysis, similar to that described in Fay et al. and Yellowlees et al. [62,66], was performed to estimate 95% CIs for the mean probability of protection, accounting for the variability in the phase 1 study. Bootstrap analysis was performed for the human data because the CIs could not easily be computed using analytical methods. The bootstrap analysis involved resampling the ZIK-101 participants blocked within dose group. Ten thousand bootstrap datasets were analyzed. For each bootstrap sample, parameters for the logistic model were sampled once from a multivariate normal distribution. This distribution had a mean equal to the original parameter estimates and a covariance matrix equal to the inverse of the Fisher Information matrix. For each dose group, the 2.5 and 97.5 percentiles from the bootstrap probabilities of protection were taken as the 95% confidence interval. Because of the small NHP sample size, bootstrapping was not used; instead, NHP parameters were sampled based on the variance covariance matrix of the original parameter estimates.

### 2.7. Bayesian LR

Bayesian LR is a modeling framework that allows for a distribution of values of interest to be generated, including model parameters and predicted values. Although the functional relationship between the predictors (in this case, NAb level) and the response (protection) is the same as for the standard LR model described above, the parameter estimates are obtained using a Markov chain Monte Carlo simulation approach.

In this analysis, Bayesian LR was conducted using the rstanarm/rstan packages, which provide R interface to the Stan C++ library for Bayesian estimation. One requirement for this modeling is the specification of a prior distribution for the parameters. Non-informative priors for both the NAb coefficient and the intercept were chosen to follow a Student’s t-distribution with 7 degrees of freedom, a mean of 0, and a scale parameter of 2.5.

## 3. Results

TAK-426 efficacy in NHPs and phase 1 trial participants’ immunogenicity results are described elsewhere [54,56].

### 3.1. TAK-426 Induced Comparable Dose-Dependent ZIKV NAb Titer GMTs in Humans (FV-Naïve) and NHPs (FVs-Naïve)

**Figure 1:**
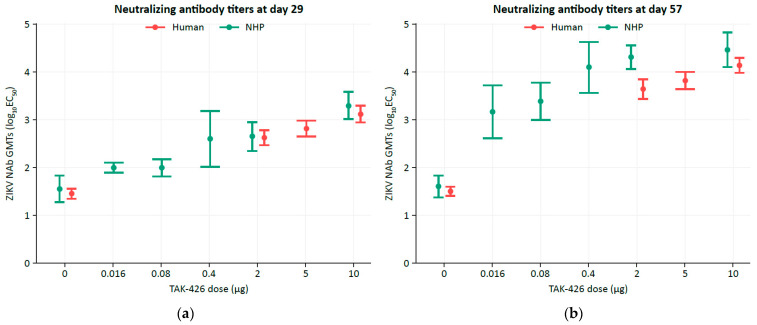
Comparison of the ZIKV NAb responses between NHPs and humans at (**a**) study days 29 and (**b**) 57. Error bars represent 95% CIs. CI, confidence interval; EC_50_, half-maximal effective concentration; NAb, neutralizing antibody; NHP, non-human primate; ZIKV, Zika virus.

**Figure 2:**
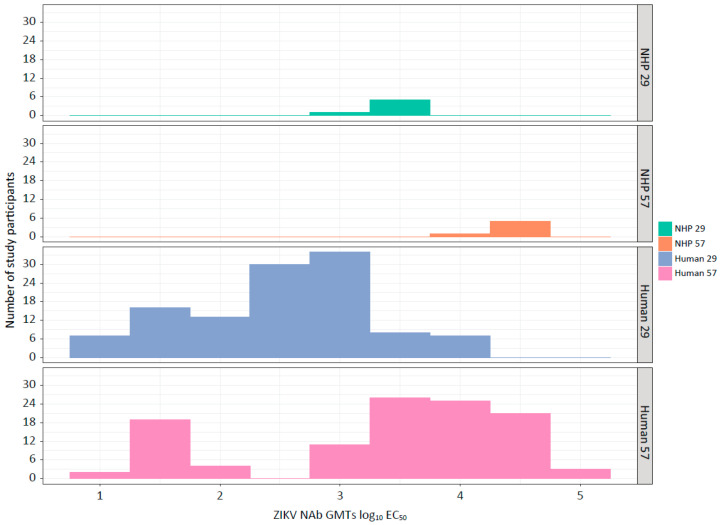
ZIKV NAb GMT distribution by species and study day for the high TAK-426 dose (10 µg). EC_50_, half-maximal effective concentration; GMT, geometric mean titer; NAb, neutralizing antibody; NHP, non-human primate; ZIKV, Zika virus.

For effective bridging between NHP and human protection, the humoral immune responses in NHPs and humans should, at the minimum, be comparable. TAK-426 induced a dose-dependent immune response that was boosted by a second immunization in both humans [56] and NHPs [54]. Table 2 and Figure 1 show the ZIKV NAb GMTs log_10_ EC_50_ and 95% CIs of across dose groups in humans (FV-naïve) and NHPs (FV-naïve) at baseline and study days 29 (1 month post dose 1) and 57 (1 month post dose 2). The magnitude of the immune response in humans and NHPs was comparable, whereby ZIKV NAb 95% CIs overlap for (i) all doses at study day 29, and (ii) for the high TAK-426 dose (10 µg selected for further development) at study day 57. Figure 2 displays histograms of ZIKV NAb GMTs for humans and NHPs at study days 29 and 57 for the high TAK-426 dose, revealing a high degree of overlap, suggesting that the prediction of human protection using the NHP NAb GMTs may be appropriate for these data.

### 3.2. Estimated Probability of TAK-426–Induced Protection in NHPs (LR Fit Using Bias Reduction)

Complete protection against ZIKV infection was achieved with the higher TAK-426 doses of 0.4 µg, 2 µg, and 10 µg at 6 weeks following vaccination. Partial protection was achieved with the lower TAK-426 doses of 0.016 µg and 0.08 µg [54].

**Figure 3:**
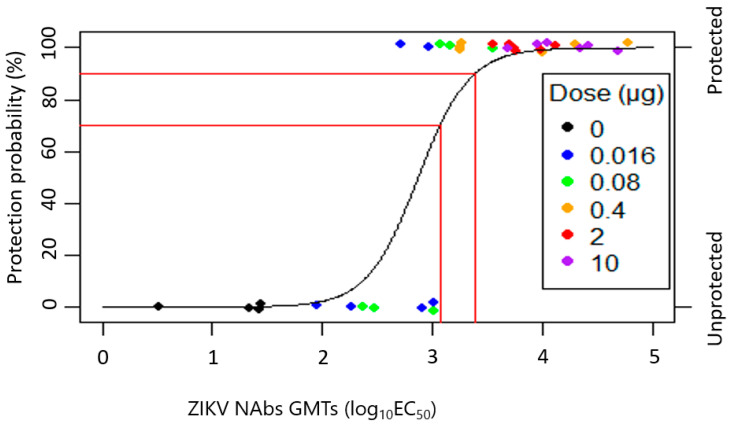
TAK-426 probability of protection versus ZIKV NAb GMTs LR at day 71 with bias reduction. To prevent overlaps in the plot, these data points have been jittered in the vertical direction; the fitted model is also displayed. The red lines show the model predicted log_10_ EC_50_ ZIKV NAb values with 70% and 90% protection. EC_50_, half-maximal effective concentration; GMT, geometric mean titer; LR, logistic regression; NAb, neutralizing antibody; ZIKV, Zika virus.

Figure 3 depicts the relationship between NHP ZIKV NAb GMTs and protection (infection), without regard to the dose. The GMTs log_10_ EC_50_ values with 70% and 90% protection are 3.07 and 3.38, respectively. For 90% protection, the 95% CI for the GMT log_10_EC_50_ is 3.07–4.55, compared to 2.71–3.25 for 70% protection. The parameters estimated from the LR model, along with 95% CIs, are presented in Table 3.

### 3.3. TAK-426 Elicited ZIKV NAbs Fulfilling the Prentice Criteria

TAK-426–induced NAb GMTs, as measured by the ZIKV RVP assay, meet the Prentice Criteria for classification as a surrogate endpoint for protection based on the NHP VE study analyses (Table 1). The TAK-426 dose was significantly related to protection (*p* = 0.0128) and to ZIKV NAb GMTs (*p* ≤ 0.0001); TAK-426-induced NAbs were significantly related to protection (*p* = 0.0145) but TAK-426 dose was not significantly related to protection after controlling for vaccine-elicited ZIKV NAb GMTs (*p* = 0.4038).

### 3.4. Estimated Probability of TAK-426–Induced Protection in Phase 1 Trial Participants, LR with Bias Reduction

Predicted probability of protection of phase 1 trial participants at different time points for each dose group was calculated and is presented in Table 4. Based on the logistic modeling of ZIKV NAb GMTs from the NHP VE study, specifically using the 90% protection threshold (GMT 3.38 log_10_ EC_50_), the probability of protection after one dose of TAK-426 against ZIKV infection in FV-naïve phase 1 participants was estimated as 30%, 46%, and 65%, and after two doses was estimated as 87%, 95%, and 98% for doses of 2 µg, 5 µg, and 10 µg, respectively.

### 3.5. Statistical Methodologies Comparison

Results from two combinations of model fits and sampling methods are presented in Table 5, which contains the probabilities of protection computed using the Bayesian model for both the posterior predictive distribution directly (Post) and the posterior predictive distribution along with bootstrapping (Boot) methods. The analysis shows that, like the LR with bias-reduction method estimates (Table 4), the Bayesian probability of protection was highest at study day 57 for all the dose groups. The Bayesian probability of protection is comparable to the LR bias-reduction model, and the 95% CIs overlap at all time points for all TAK-426 doses.

The Bayesian approaches, which differ only in their sampling methods, resulted in nearly identical protection estimates. The LR with bias-reduction method estimates differ slightly from those obtained using the Bayesian approach, with slightly higher estimates in the lower range and lower estimates in the higher range of protection probabilities. This effect is a direct result of computing logistic curves from their different parameter estimates: 4.7 (Bayesian) versus 4.3 (bias reduction) as the estimated coefficient for the NAb term.

As described above, based on LR fit with bias reduction, a probability of 90% protection in humans was expected with ZIKV NAb GMTs ≥ 3.38 log_10_ EC_50_, the TAK-426 preliminary surrogate of protection threshold. These results were confirmed using an alternative Bayesian LR approach, where a probability of protection of 90% is associated with a GMT value of 3.4 log_10_ EC_50_.

## 4. Discussion

Using the statistical framework presented herein to derive a surrogate of protection for TAK-426, a probability of 90% protection in NHPs and humans (FV-naïve adults) is expected with ZIKV NAb GMTs of ≥3.38 log_10_ EC_50._ To test the robustness and confirm the validity of the TAK-426 LR with bias-reduction results, the Prentice Criteria and a Bayesian LR model were applied. TAK-426–elicited ZIKV NAbs in NHPs that fulfilled the Prentice Criteria (Table 1) and the probability of protection, estimated by the Bayesian LR, were similar to the LR with bias-reduction model.

TAK-426 passive immunization studies of naïve mice with TAK-426–immune sera (mouse and NHP) or purified immunoglobulin G from TAK-426–immune sera (human) have demonstrated a strong inverse correlation between ZIKV NAb GMTs and protection against virus challenge (Baldwin et al. 2018 and unpublished data) [53]. Passive immunization studies in naïve NHPs may provide further support for the use of TAK-426–induced ZIKV NAbs as a surrogate of protection.

A major limitation faced by ZIKV vaccine developers is the lack of an immune marker of protection following ZIKV natural infection such as the one reported for chikungunya virus [67,68] used for FDA licensure [68] of IXCHIQ and recommendations [69] following the US Food and Drug Administration’s Accelerated Approval pathway. In the absence of published longitudinal data generated by ZIKV long-term cohort studies, clinical trials could fill this critical data gap. A controlled human Zika infection challenge model study may identify a ZIKV or vaccine-induced immune marker [70]. Furthermore, a retrospective analysis among phase 3 efficacy dengue participants exposed to the Zika outbreak in Latin America [71] could also determine a level of ZIKV NAbs that correlates with ZIKV natural infection risk. In the meantime, seroepidemiological studies are useful to support our model results by indicating that the magnitude and kinetics of TAK-426–induced ZIKV NAbs are similar to those elicited by ZIKV natural infection [57]. Another limitation is the extent to which this modeling approach could be applied to populations with pre-existing FV immunity, either from natural infection or vaccination. Although heterologous flavivirus vaccination (against Yellow fever, Japanese encephalitis, West Nile, and tick-borne encephalitis viruses) did not impact TAK-426 VE in NHPs against viremia after ZIKV challenge 8–12 months post-PIZV vaccination, it had a variable impact on ZIKV neutralizing antibody titers [55]. The potential role of previous FVs infections, DENV and/or dengue vaccination in particular, are to be first assessed in NHPs and subsequently among humans in future studies.

An underlying assumption for this Zika vaccine clinical development path is that if the vaccine protects against infection in NHPs, it will then protect against infection in humans and therefore ought to prevent severe clinical presentations such as CZS and Guillain–Barré syndrome. As for CZS, such protection should be assessed in a NHP fetal model whereby TAK-426 protection, against ZIKV-induced CZS, is demonstrated. One consideration for the work presented in this manuscript is that protection in the NHP model was stringently defined as protection from infection (serum vRNA below the lower limit of quantitation for all days tested post challenge), and it is unknown how closely this limit of quantification translates to human clinical efficacy (e.g., preventing infection and birth defects). Notably, a study of NHPs suggested that sterilizing immunity in the mother may not be required for protection against CZS [72]; therefore, even under non-sterilizing immunity conditions, a ZIKV vaccine could, in principle, protect against CZS. Regardless, it is encouraging that two 10 µg TAK-426 vaccinations elicited sterilizing immunity and long-term protection in rhesus macaques [72].

It is generally assumed that ZIKV natural infection induces life-long protective immunity, although a recent genome diversity and antibody response analysis seems to provide some evidence for the existence of ZIKV mild/asymptomatic reinfections [73]. In any case, 1-year duration of protection in NHPs suggests that, despite a decline in NAbs from study day 57 to 85 [54], the predicted high efficacy of TAK-426 in humans could be sustained for at least 1 year. Similarly, despite the decline in human NAbs observed 6 months post dose 2, the 2-year duration of TAK-426-induced steady-state NAbs [57] may indicate sustained predicted efficacy for at least 2 years. Of note, as mentioned above, the decline and steady state of TAK-426-induced NAbs are comparable to those induced by ZIKV natural infection [57]. The limited long-term protection data on TAK-426 should be complemented with longer follow-ups and further characterization of the quality and duration of the antibody response (e.g., B-cell memory response, and T cell-mediated immune response) in future clinical studies.

Nonetheless, under the current ZIKV transmission conditions (characterized by low levels of circulation and the absence of major outbreaks), the use of TAK-426–elicited ZIKV NAbs as a surrogate of protection in the pathway for licensure is supported by published data on TAK-426 in humans and animal models and has been discussed with regulators. These initial proof-of-concept experiments were performed utilizing non-validated fit-for-purpose assays (Zika RVP, RT-qPCR) and phase 1 clinical trial material (CTM-1). The experiments and analyses are to be repeated utilizing CTM from the phase 2 manufacturing process (CTM-2) and validated Zika RVP and RT-qPCR assays. These phase 2 experiments and analyses will establish the threshold to be used as the surrogate of protection primary endpoint in phase 3. Herein, we provided a statistical framework to predict the probability of protection for human phase 1 trial participants vaccinated with TAK-426 in the absence of direct VE measurements, using animal data under a single-correlate model. TAK-426 predicted VE is to be confirmed in a post-licensure study.

## Figures and Tables

**Table 1:** Application of the Prentice Criteria to ZIKV NAbs from the NHP VE study.

Criterion	Application
1: Protection is significantly related to vaccine dose in the animal study	Fit LR with protection status as defined by vRNA as the response and log_10_ dose as the independent variable
2: ZIKV NAbs are significantly related to vaccine dose in the animal study	Fit linear regression with ZIKV NAb GMTs as the response and log_10_ dose as the independent variable
3: ZIKV NAbs are significantly related to protection in the animal study	Fit LR with protection status as the response and ZIKV NAb GMTs as the independent variable
4: The full effect of the vaccine on protection is explained by ZIKV NAbs	Fit a similar model as in step 3, with the addition of a term for vaccine log_10_ dose. Determine that the log_10_ dose term does not contribute significantly to the model fit

LR, logistic regression; NAb, neutralizing antibody; NHP, non-human primate; VE, vaccine efficacy; ZIKV, Zika virus.

**Table 2:** Human and NHP NAb GMTs (95% CI) by RVP EC_50_, over time.

	N	TAK-426 Dose, µg	GMT (95% CI)		
			Baseline	Study Day 29	Study Day 57
Humans	28	0	34 (27–44)	28 (22–36)	31 (25–40)
	25	2	37 (29–47)	408 (274–607)	3701 (2330–5878)
	29	5	34 (28–40)	688 (468–1010)	6977 (4663–10,438)
	30	10	48 (41–56)	1310 (875–1961)	13,604 (9560–19,359)
NHPs	4	0	45 (25–79)	35 (19–67)	40 (24–67)
	6	2	47 (29–75)	437 (218–876)	20,145 (11,363–35,714)
	0	5	N/A	N/A	N/A
	6	10	57 (39–84)	1959 (1014–3785)	28,892 (12,607–66,212)

CI, confidence interval; EC_50_, half-maximal effective concentration; GMT, geometric mean titer; N/A, not applicable; NAb, neutralizing antibody; NHP, non-human primate.

**Table 3:** Probability of protection versus ZIKV NAb GMTs, logistic regression with bias reduction.

Parameter	Estimate	95% LCL	95% UCL
Intercept	−12.39	−22.87	−1.91
Slope	4.31	0.85	7.77
Hosmer–Lemeshow goodness-of-fit test statistic	1.71	N/A	N/A
Hosmer–Lemeshow test *p*-value	0.99	N/A	N/A
Area under the curve	0.98	N/A	N/A

LCL, lower confidence limit; N/A, not applicable; NAb, neutralizing antibody; UCL, upper confidence limit.

**Table 4:** Phase 1 trial–estimated probability of protection of TAK-426 against ZIKV in FV-naïve cohort, logistic regression with bias reduction.

	Estimated Probability of Protection, against ZIKV, and 95% CI		
	Baseline	Day 29	Day 57
Placebo	1 (0–40)	0 (0–36)	0 (0–33)
TAK-426 2 µg	1 (0–36)	30 (13–66)	87 (63–96)
TAK-426 5 µg	0 (0–39)	46 (25–74)	95 (71–99)
TAK-426 10 µg	1 (0–43)	65 (42–84)	98 (80–100)

CI, confidence interval; FV, flavivirus; ZIKV, Zika virus.

**Table 5:** Estimated probabilities of protection and credible intervals, Bayesian logistic regression (probability level = 0.95).

Study Day	TAK-426 Dose, µg	Probability (Protection) Post, %	Lower Post, %	Upper Post, %	Probability (Protection) Boot, %	Lower Boot, %	Upper Boot, %
29	2	21	3	55	21	2	57
	5	42	13	72	42	11	75
	10	74	47	91	74	41	94
57	2	95	80	100	95	78	100
	5	99	89	100	98	89	100
	10	100	95	100	100	95	100

## Data Availability

The datasets from the clinical study NCT03343626, will be made available within 3 months from initial request to researchers who provide a methodologically sound proposal. The datasets used and/or analyzed during the current study are available from the corresponding author on reasonable request. The data will be provided after its de-identification, in compliance with applicable privacy laws, data protection, and requirements for consent and anonymization.
